# Effective cancer immunotherapy by natural mouse conventional type-1 dendritic cells bearing dead tumor antigen

**DOI:** 10.1186/s40425-019-0565-5

**Published:** 2019-04-08

**Authors:** Stefanie K. Wculek, Joaquín Amores-Iniesta, Ruth Conde-Garrosa, Sofía C. Khouili, Ignacio Melero, David Sancho

**Affiliations:** 1 Immunobiology Laboratory, Centro Nacional de Investigaciones Cardiovasculares (CNIC), Melchor Fernández Almagro, 3, 28029 Madrid, Spain; 2 Division of Immunology and Immunotherapy, Center for Applied Medical Research (CIMA), University of Navarra, and Instituto de Investigación Sanitaria de Navarra (IdISNA), Pamplona, Spain; 3 University Clinic, University of Navarra and Instituto de Investigación Sanitaria de Navarra (IdISNA), Pamplona, Spain; 4 Centro de Investigación Biomédica en Red Cáncer (CIBERONC), Madrid, Spain

**Keywords:** Conventional dendritic cells, cDC1, Cancer immunotherapy, Vaccination, Cell-associated antigen, Cross-presenting dendritic cells, Immunogenic cell death

## Abstract

**Background:**

The manipulation of dendritic cells (DCs) for cancer vaccination has not reached its full potential, despite the revolution in cancer immunotherapy. DCs are fundamental for CD8+ T cell activation, which relies on cross-presentation of exogenous antigen on MHC-I and can be fostered by immunogenic cancer cell death. Translational and clinical research has focused on in vitro-generated monocyte-derived DCs, while the vaccination efficacy of natural conventional type 1 DCs (cDC1s), which are associated with improved anti-tumor immunity and specialize on antigen cross-presentation, remains unknown.

**Methods:**

We isolated primary spleen mouse cDC1s and established a protocol for fast ex vivo activation and antigen-loading with lysates of tumor cells that underwent immunogenic cell death by UV irradiation. Natural tumor antigen-loaded cDC1s were transferred and their potential for induction of endogenous CD8+ and CD4+ T cell responses in vivo, cancer prevention and therapy were assessed in three grafted cancer models. Further, we tested the efficacy of natural cDC1 vaccination in combination and comparison with anti-PD-1 treatment in two “wildtype” tumor models not expressing exogenous antigens.

**Results:**

Herein, we reveal that primary mouse cDC1s ex vivo loaded with dead tumor cell-derived antigen are activated and induce strong CD8+ T cell responses from the endogenous repertoire upon adoptive transfer in vivo through tumor antigen cross-presentation. Notably, cDC1-based vaccines enhance tumor infiltration by cancer-reactive CD8+ and CD4+ T cells and halt progression of engrafted cancer models, including tumors that are refractory to anti-PD-1 treatment. Moreover, combined tumor antigen-loaded primary cDC1 and anti-PD-1 therapy had strong synergistic effects in a PD-1 checkpoint inhibition susceptible cancer model.

**Conclusions:**

This preclinical proof-of-principle study is first to support the therapeutic efficacy of cancer immunotherapy with syngeneic dead tumor cell antigen-loaded mouse cDC1s, the equivalents of the human dendritic cell subset that correlates with beneficial prognosis of cancer patients. Our data pave the way for translation of cDC1-based cancer treatments into the clinic when isolation of natural human cDC1s becomes feasible.

**Electronic supplementary material:**

The online version of this article (10.1186/s40425-019-0565-5) contains supplementary material, which is available to authorized users.

## Background

Cytotoxic CD8+ T lymphocytes (CD8+ CTLs) are key effector cells that recognize and eliminate tumor cells and therefore preferential targets for improving cancer immunotherapy [1]. However, cancer-reactive CD8+ T cells can become dysfunctional or exhausted limiting their efficacy [2]. Immune checkpoint inhibitors, such as anti-PD-1 or anti-CTLA4, have achieved unprecedented success in the treatment of various cancers by reinvigorating exhausted CD8+ CTLs. Unfortunately, their clinical benefits remain limited and it is crucial to find additional strategies to increase basal anti-tumor CD8+ T cell immunity [3, 4].

Dendritic cell (DC) vaccines may elicit and improve anti-cancer CD8+ T cell immunity [5, 6]. However, the use of DCs for tumor immunotherapy has been limited so far, being the only FDA-approved therapy the blood antigen (Ag)-presenting cell-based vaccine Sipuleucel-T for metastatic castration-resistant prostate cancer [7]. Currently, most clinical efforts focus on human blood monocyte-derived DCs (moDCs) cultured for several days with GM-CSF and IL-4, loaded with various Ags and stimulated with proinflammatory adjuvants [6, 8–10]. Numerous clinical trials are ongoing that test the efficacy of moDC-based vaccination against different types of cancer as single agents or combination therapies. Combination of DC vaccination and immune checkpoint inhibitors appears especially promising, as both target enhanced mobilization and activity of anti-cancer CD8+ T cells [5, 6, 11]. In general, adoptive transfer of ex vivo-treated DCs to cancer patients demonstrated an excellent safety profile, but the efficacy did not meet the expectations yet [12].

The advance in understanding of distinct DC subset functions calls for the development of next-generation DC vaccines, by using primary DC subsets from patient blood that excel in induction of anti-cancer CD8+ T cell immunity [8, 10, 11, 13, 14]. Circulating human DC subsets comprise two types of conventional (c) DCs, cDC1s (BDCA3+ cDCs) and cDC2s (CD1c+ cDCs) as well as plasmacytoid DCs (BDCA2+ BDCA4+ pDCs), which specialize on different functions [15]. Both human pDCs and cDC2s from blood have been used as basis for next-generation DC vaccines [16, 17], which led to an ongoing phase III clinical trial (ClinicalTrials.gov identifier: NCT02993315).

Human cDC1s (DNGR-1/CLEC9A+, XCR1+) and their mouse equivalents (DNGR-1/Clec9a+, Xcr1+, also expressing CD8 in lymphoid organs) are strong inducers of CD8+ T cell responses due to their superior capacity for uptake of dying or dead cell material and processing of cancer cell-associated Ags for cross-presentation [18–22]. Moreover, cDC1s are the main cellular source of IL-12 [23], a fundamental cytokine for anti-cancer CD8+ CTL activation [24]. Those functional traits support the notion that cDC1s are the superior DC subset for induction of anti-tumor immunity [25, 26]. Indeed, Batf3-dependent cDC1s are needed to mount anti-tumor CD8+ T cell responses at baseline [25] or upon poly I:C therapy [27] and are indispensable for efficacy of immune checkpoint blockade [28, 29]. Moreover, cDC1s are critical for the transport of intact tumor-Ag to lymph nodes (LNs) to elicit anti-tumor T cell activation [29, 30] and the secretion of CXCL9/10 by tumor-infiltrating cDC1s is crucial for the recruitment of CD8+ T cells into tumors [31]. Moreover, a variety of studies show that presence of cross-presenting BDCA3+ cDC1s or their gene signatures in the tumor correlates with enhanced T cell infiltration, improved prognosis and survival of cancer patients [26, 30, 32, 33].

Despite these evidences, natural cDC1s have not been previously tested as a syngeneic vaccine in cancer therapy. We herein demonstrate the feasibility and efficacy of an anti-cancer treatment based on adoptive transfer of the natural cross-presenting mouse cDC1 subset loaded ex vivo with an autologous whole tumor cell lysate (TCL) obtained after induction of immunogenic cell death (ICD) by UV irradiation. The cDC1 vaccine induces substantial anti-tumor CD8+ T cell responses from the endogenous repertoire in vivo, that depend on their cell-autonomous cross-presentation potential. Notably, this treatment fosters tumor-reactive CD8+ and CD4+ T cell presence in tumors and tumor-draining LNs (tdLN), limiting tumor progression of three different mouse cancer models, of which two do not express exogenous or dominant Ags. TCL-loaded cDC1 administration profoundly improves anti-PD-1 therapy in an immune checkpoint antibody sensitive model and, notably, is also effective for treatment of tumors that are refractory to anti-PD-1. Therefore, we provide valuable pre-clinical information on the efficacy of therapeutic cDC1-based anti-cancer vaccination for the development of next-generation DC vaccines [8].

## Methods

### Mice

Mouse colonies were bred at the CNIC under specific pathogen-free conditions. Wildtype mice were in C57BL/6 background and 6–10-weeks old females used for all experiments. B6.C-*H2-K*^*bm1*^ (B6.C-H2-K^bm1^/ByJ or C57BL/6^H2Kbm1^) mice were kindly provided by Caetano Reis e Sousa (The Crick Institute, London, UK) and OT-I transgenic mice (C57BL/6-Tg (TcraTcrb)1100Mjb/J) crossed with B6-SJL (Ptprca Pepcb/BoyJ) mice expressing the CD45.1 allele were both from The Jackson Laboratory (Bar Harbor, ME, USA).

### Tissue dissociation for cell isolation

Spleen and inguinal lymph nodes (iLNs) were harvested in R10 medium [RPMI Medium 1640 (Gibco®) with 10% heat-inactivated Fetal Bovine Serum (hi-FBS), 50 μM β-Mercaptoethanol (both Sigma), 2 mM L-Glutamine, 100 U/mL Penicillin and Streptomycin (100 μg both Lonza), 0.1 mM NEAA, 1 mM Sodium Pyruvate, 1 mM HEPES (all from HyClone™)]. Spleen was digested for 10 min with 0.25 mg/ml Liberase TL (Roche) and 50 μg/ml DNaseI (Sigma Aldrich). Tumors were minced and incubated for 30 min in HBSS (Gibco®) with 0.5 mg/ml Collagenase IV (Sigma) and 50 μg/ml DNAseI shacking at 37 °C. Tissues were squeezed through a 70 μm cell strainer (Corning), re-filtered through a 40 μm cell strainer and spleen subjected for 5 min to Red Blood Cell Lysis Buffer (Sigma).

### Purification and adoptive transfer of CD8+ spleen DCs

For cDC1 expansion, 2.5 × 10^6^ B16-Flt3L cells in 100 μl PBS were inoculated subcutaneously into both flanks of wildtype or C57BL/6^H2Kbm1^ mice and spleens harvested 9–11 days thereafter or naïve mice used. Spleen CD8+ cDC1 cells were isolated using the mouse CD8+ Dendritic Cell Isolation Kit (Order no. 130–091-169) using MACS® columns and autoMACS™ Running Buffer according to manufacturer’s instructions (Miltenyi Biotec). In brief, spleen single cell suspensions were subjected to negative selection that depletes T, B and NK cells, followed by positive selection of CD8a DCs. Purified cDC1s were cultured in round-bottom 96-well plates (Corning) at 2 × 10^5^ cDC1s/200 μl R10 medium for 1 h at 37 °C in 5% CO_2_ together with (as specified for experiments): 20 μg/ml poly I:C LMW (InVivoGen), 20 μg/ml Hiltonol (kindly provided by Andres Salazar from Oncovir), 20 μg/ml BO112 (Bioncotech Therapeutics), 20 μg/ml endotoxin-free soluble OVA protein (EndoGrade from Hyglos), and/or B16-OVA, B16/F10 or MC38 TCL at a ratio of 1 DC to 2 tumor cells. cDC1s were washed with R10 and, when incubated with TCL, re-purified using MACS® columns. Cells were kept in culture for 4 h for analysis of CD86 and MHC-II induction, immediately added to naive OT-I cells or 2–10 × 10^5^ cDC1s injected intravenously (100 μl PBS, Gibco®) or intradermally (50 μl PBS) into mice. Anti-PD-1 (clone RMP1–14 from BioXCell) was administered intraperitoneal at 100 μg/mouse in 100 μl PBS.

### OT-I CD8+ T cell assays

Total spleen CD8+ OT-I cells for in vivo assays were purified from CD45.1 OT-I transgenic mice by negative selection as follows: 30 min incubation of spleen single cell suspensions with biotinylated antibodies (all BD Biosciences) for anti-mouse CD16/CD32 (clone 2.4G2), CD4 (clone GK1.5), B220 (clone RA3-6B2), CD11c (clone HL3), CD11b (clone M1/70), Gr-1 (clone RB6-8C5) and I-A/I-E (MHC-II, clone 2G9) at 4 °C shaking, washing, 20 min incubation with Streptavidin MicroBeads in autoMACS™ Running Buffer and magnetic separation using LD columns (all Miltenyi Biotec) according to manufacturer’s instructions. For ex vivo proliferation assay, naive CD8+ CD44- CD62L+ OT-I cells were isolated by flow cytometric sorting using the SY3200 Cell Sorter (Sony Biotechnology). Cells were labeled with CellTrace™ Violet Cell Proliferation Kit (Thermofisher, Molecular Probes) according to manufacturer’s instructions. 1-2 × 10^5^ total labeled OT-I cells in 100 μl PBS were injected intravenously into mice. Naive OT-I cells were cultured for 3 days with pre-treated cDC1s in R10 medium (1:1 ratio) in round-bottom 96-well plates at 37 °C in 5% CO_2_ followed by flow cytometric analysis of proliferation by dilution of the CellTrace™ Violet dye.

### Fluorescent staining, flow cytometry and cell sorting

Single cell suspensions of cDC1s, spleen, iLN and tumors or cultured OT-I cells were incubated for 20 min at 4 °C in PBS with 2% hi-FBS and 0.5 mM EDTA (Sigma) with FcR block anti-mouse CD16/CD32 (2.4G2, Tonbo Biosciences) and a mix of the following fluorochrome-conjugated antibodies: anti-mouse CD45.1 (clone A20), CD44 (clone IM7), SIRPα (clone P84) and CD62L (MEL-14) from eBioscience™, CD11c (clone HL3), CD11b (clone M1/70), and I-A/I-E (MHC-II, clone 2G9) from BD Biosciences, CD3 (clone 17A2), CD86 (clone GL-1) and PD-1 (clone J43.1) from Tonbo Biosciences, CD8 (clone 53–6.7) from BioLegend, XCR1 (clone REA707), CD205 (clone NLDC-145), Clec9A (clone 7H11) and CD24 (clone M1/69) from Miltenyi Biotec. When indicated, cells were beforehand incubated with Allophycocyanin-conjugated OVA H-2Kb (257-SIINFEKL-264) dextramer (Immunodex, catalogue number JD2163) or a mix of allophycocyanin-conjugated OVA-specific MHC-II tetramers (I-A(b) 329-AAHAEINEA-337, I-A(b) 328-HAAHAEINEA-337 and I-A(b) 325-QAVHAAHAEIN-325; all from the NIH Tetramer Facility at Emory University) for 20 min at room temperature. Hoechst 332558 (Sigma) or SYTOX Green (Thermofisher) was used to exclude dead cells. The LSRFortessa cell analyzer running FACSDiva software (BD Biosciences) and FlowJo Version 10 or FCS Express 6 Plus software was used to record and analyze data.

### Tumor cell culture, inoculation, in vivo analysis and HMGB1 ELISA

B16/F10 (a kind gift from I. Malanchi, The Crick Institute, London, UK), B16-OVA (a kind gift from L. Chen, Yale University, New Haven, CT), MC38 (purchased from the ATCC) [28] and B16-Flt3L cells (kindly provided by G. Dranoff, Harvard University, Boston, MA) [34] were cultured at 37 °C in 5% CO_2_ in R10 medium. The B16-OVA cell line expresses a truncated and non-secreted OVA protein without the signal peptide as a fusion protein with EGFP-C1, as previously described for mouse embryonic fibroblasts [35]. All cell lines were tested for absence of mycoplasma using the MycoAlert PLUS Mycoplasma Detection Kit (Lonza) according to manufacturer’s instructions. Tumor cells were detached (5 mM EDTA/PBS) before reaching confluence and 1 × 10^6^ B16-OVA or 0.5 × 10^6^ B16/F10 cells inoculated intradermal or 0.5 × 10^6^ MC38 cells inoculated subcutaneous in 50 μl PBS into the shaved right flank of wildtype mice. Tumor size was measured three times weekly using a digital caliper (Ratio), calculated as the product of orthogonal diameters and is displayed in mm^2^. Tumor-bearing mice were monitored daily and sacrificed to determine the survival curve when signs of adverse effects (pain, apathy, dehydration, necrotic tumor) were observed or the humane endpoint (tumor size diameter 1.7 cm) reached. Pictures were taken at indicated time points using a digital camera. For HMGB1 ELISA, 0.5 × 10^6^ cancer cells were UV-irradiated or treated with doxorubicin (25μM), brefeldin A (50μM) or mitomycin C (30μM, all from Sigma) and cultured in 1 ml R10 for 18 h, supernatant harvested and solid components removed by centrifugation. HMGB1 was measured using the HMGB1 ELISA (IBL international GmbH) following manufacturer’s instructions.

### Tumor cell lysate preparation

B16-OVA, B16/F10 or MC38 tumor cells were adjusted to 4 × 10^6^ cells/ml in R10 medium, 1.5 ml per well plated in 6-well plates (Corning) and treated with 300 mJ/cm^2^ UV irradiation using a Stratalinker UV Crosslinker 1800 (Stratagene). Cells were cultured for 16-24 h at 37 °C in 5% CO_2_, subjected to 3 freeze (− 80 °C) / thaw (37 °C) cycles of minimum 30 min each and passed through a 40 μm cell strainer before addition to cDC1s at a ratio of 1 DC to 2 tumor cells. B16-OVA TCL was twice washed with R10 and centrifuged at full-speed to remove soluble components and obtain “washed B16-OVA TCL”.

### Spleen, tdLN and tumor re-stimulation

For intracellular IFNγ staining, single cell suspensions were ex vivo re-stimulated with 2 μM OVA_257–264_ peptide (SIINFEKL, GenScript), OVA_323–339_ peptide (ISQAVHAAHAEINEAGR, GenScript)-loaded or B16-OVA TCL-loaded antigen-presenting cells (APCs) for 2 h in R10 at 37 °C in 5% CO_2_ followed by 5 μg/mL Brefeldin A (Sigma Aldrich) treatment for 4 h. Cells were labeled with indicated surface-staining antibodies, fixed with 4% PFA (Thermofisher), permeabilized with 1% Bovine Serum Albumin, 0.1% Saponin, 0.02% Sodium Azide (all Sigma) in PBS and stained with Allophycocyanin-conjugated anti-mouse IFNγ antibody (clone XMG1.2, eBioscience™). APCs were generated from bone marrow cells, harvested by flushing the tibia and femur and a red blood cell lysis. Then, bone marrow was cultured in R10 with 20 ng/ml murine GM-CSF (Peprotech) for 7 days; floating cells were harvested and incubated with either 5 μM OVA_323–339_ peptide or B16-OVA TCL for 8 h followed by addition of 100 ng/ml LPS-EK (InvivoGen) for another 12 h.

### RNA isolation and quantitative PCR

Total RNA was extracted with the RNeasy Micro Kit (Qiagen) and reverse transcribed using the High Capacity cDNA Reverse Transcription Kit with random hexamers (Applied Biosystems®) following manufacturer’s instructions. Quantitative PCR was performed using the GoTaq® qPCR Master Mix (Promega) in a 7900HT Fast Real-Time PCR System (Applied Byosystem®). 2^-ΔCt^ mRNA expression values of mouse *Ifnb1*, *Il12b*, *CD40* and *CCR7* were calculated relative to expression of *Actb.* Primers (Sigma):

- *Actb*-sense (5′)–GGCTGTATTCCCCTCCATCG–(3′),
- *Actb*-antisense (5′)–CCAGTTGGTAACAATGCCATGT–(3′);
- *Ifnb1-*sense (5′)–TCAGAATGAGTGGTGGTTGC–(3′),
- *Ifnb1-*antisense (5′)–GACCTTTCAAATGCAGTAGATTCA–(3′);
- *Il12b*-sense (5′)–GGAAGCACGGCAGCAGAATA–(3′),
- *Il12b*-antisense (5′)–AACTTGAGGGAGAAGTAGGAATGG–(3′);
- *CD40*-sense (5′)–TTGTTGACAGCGGTCCATCTA–(3′),
- *CD40*-antisense (5′)–GCCATCGTGGAGGTACTGTTT–(3′);
- *CCR7*-sense (5′)–TGTACGAGTCGGTGTGCTTC–(3′),
- *CCR7*-antisense (5′)–GGTAGGTATCCGTCATGGTCTTG–(3′).

### Statistical analysis

Data analyses employed GraphPad Prism version 7.0c. Data are presented as mean ± standard error of the mean, individual values, staircase graph with ticks, ‘scatter plot with box & whiskers’ and/or ‘scatter plot with column bar’ graph and were analyzed using Two-tailed Student’s *t*-test (Paired or unpaired according to experimental setting), One-way ANOVA and Tukey post hoc test, Mantel-Cox test and Two-way ANOVA. All experiments were repeated at least twice and either representative experiments or pooled data from several experiments are shown as indicated in the figure legends. Mice were allocated randomly in different experimental groups, but no blinding or randomization strategy was used. No animals were excluded from analysis, unless they had wounds from fighting/over-grooming. All *n* values represent biological replicates (different mice, primary cell preparations or in vitro experiments). Differences were considered significant when *P* < 0.05 and are indicated as ns, not significant, *P < 0.05, **P < 0.01, ***P < 0.001.

## Results

### Adoptive transfer of antigen and adjuvant-treated natural mouse cDC1s promotes CD8+ T cell immunity

Comparative transcriptional and functional analyses have established that mouse spleen CD8α+ cDC1s are the equivalents of human circulating BDCA3+ cDC1s [18–21]. Therefore, we used naturally occurring mouse spleen CD8α+ cDC1 in order to test the suitability of cDC1s as basis for effective cancer therapy. Human anti-cancer DC vaccines are based on autologous DCs obtained from the cancer patient [36]. To reproduce this situation in our pre-clinical setting while expanding cDC1s, mice were grafted with B16 melanomas that secrete FMS-like tyrosine kinase 3 ligand (FLT3L) (B16-FLT3L) [34]. CD11c+ MHC-II+ CD8α+ cDC1s isolated from the spleen of both steady-state and B16-FLT3L tumor-bearing donor mice showed similar patterns of surface markers, including high XCR1, CD205, Clec9A and CD24, as well as no/low CD11b and SIRPα expression (Additional file 1: Figure S1), suggesting no major phenotypic alteration of cDC1s in B16-FLT3L tumor-bearing mice.

**Fig. 1:**
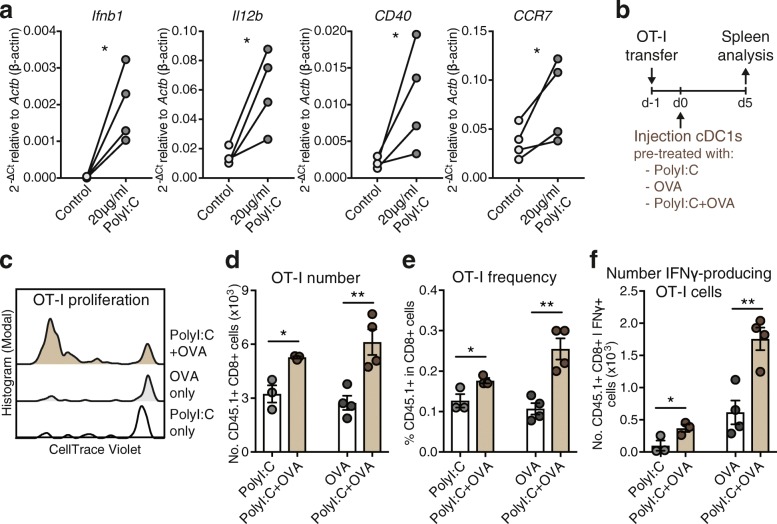
Adoptive transfer of Ag and adjuvant-treated natural mouse cDC1s promotes CD8+ T cell immunity. **a** Quantitative PCR analyses of mRNA levels of *Ifnb1* (IFNβ protein), *Il12b* (IL12-p40 protein), *CD40* and *CCR7* relative to *Actb* (β-actin protein) of spleen cDC1s freshly isolated from B16-FLT3L tumor-bearing mice and treated with 20 μg/ml poly I:C (InvivoGen) for 1 h ex vivo, *n* = 4 spleen cDC1 preparations. *P < 0.05 by Ratio paired Student’s *t* test. Ct: Cycle threshold, −ΔCt = −(Ct [gene of interest] - Ct [*Actb* internal control gene]). **b** Schematic representation of experimental setup for data shown in **c**-**f**. Spleen cDC1s from B16-FLT3L tumor-bearing mice were cultured with 20 μg/ml poly I:C and/or 20 μg/ml soluble OVA protein for 1 h, washed and 2 × 10^5^ cDC1s injected intravenously into CD45.2+ recipient mice that had been adoptively transferred with 1-2 × 10^5^ CellTrace-Violet (CV)-labelled CD45.1+ OT-I CD8+ T cells one-day prior. **c** Representative flow cytometric analysis of OT-I T cell proliferation via CV-dilution in the spleen at day 5, gated on CD45.1+ CD8+ OT-I cells. **d**-**f** Flow cytometric quantification of **d** total OT-I cell number, **e** OT-I frequency in CD8+ cells and **f** number of IFNγ-producing OT-I cells after re-stimulation with OVA_257–264_ peptide in the spleen at day 5 post-cDC1 injection. One representative of 2 independent experiments (*n* = 3–4 mice/group/experiment) is shown. *P < 0.05, **P < 0.01 by Student’s *t* test

To address the efficacy of naturally occurring cDC1s for the induction of CD8+ T cell immunity upon transfer in vivo, we treated cDC1s ex vivo with soluble Ovalbumin (OVA) as Ag and poly I:C, a synthetic double-stranded RNA adjuvant that binds Toll-like receptor (TLR) 3, which is selectively expressed by mouse and human cDC1s and promotes their activation and function [20, 27]. Indeed, poly I:C treatment ex vivo rapidly activated natural mouse cDC1s, inducing transcription of cytokines and surface receptors involved in CD8+ T cell priming (Fig. 1a). Natural cDC1s were then intravenously (IV) injected into syngeneic recipient mice that were previously adoptively transferred with OT-I OVA-specific CD8+ T cells (Fig. 1b). Adoptive transfer of natural cDC1s shortly exposed to OVA and poly I:C ex vivo led to increased OT-I proliferation, frequencies in CD8+ T cells and total numbers, as well as augmented IFNγ producing OT-I cells after specific MHC-class I OVA_257–264_ peptide re-stimulation, as compared with mice transferred with cDC1s pre-treated only with OVA or poly I:C (Fig. 1c-f). Hence, natural spleen cDC1s treated ex vivo with Ag and adjuvant for just 1 h strongly activate CD8+ T cells in vivo in an adjuvant and Ag-dependent fashion.

### Tumor cell lysate-loaded cDC1s generate optimal CD8+ T cell activation in vivo

**Fig. 2:**
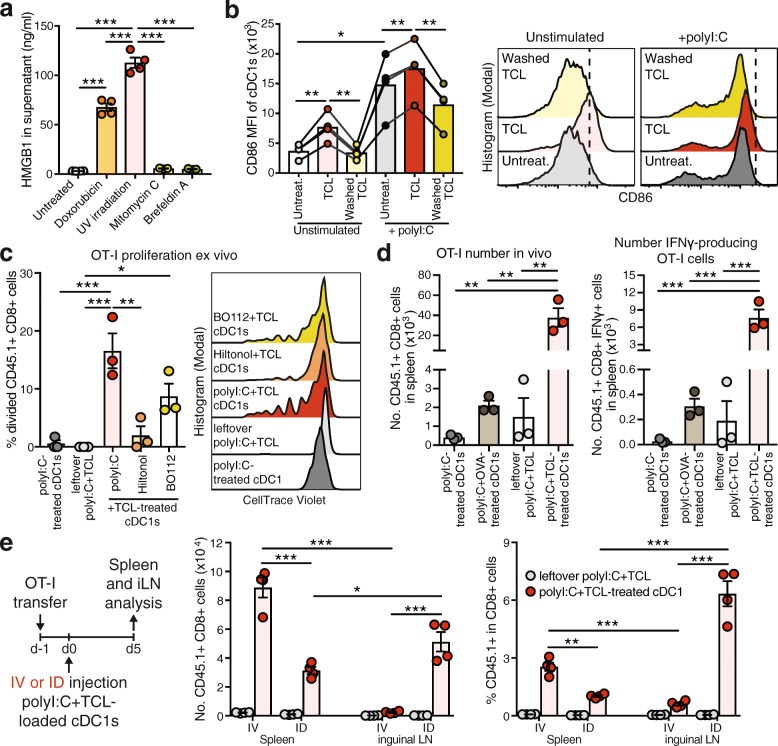
TCL induces cDC1 activation and TCL-loaded cDC1s generate optimal CD8+ T cell activation in vivo. **a** HMGB1 content measured by ELISA in supernatants of B16-OVA cells treated with indicated agents (n = 3–4). **b** Quantification (left) and representative histograms (right) of CD86 expression on untreated cDC1s (Untreat.), cDC1s treated either with B16-OVA tumor cell lysate (TCL) or washed B16-OVA TCL containing only cellular components +/− 20 μg/ml poly I:C. After 1 h, cDC1s were washed and cultured for 4 h followed by flow cytometric analysis. Combined data of 4 independent experiments. MFI, mean fluorescent intensity. **c** Spleen cDC1s were cultured with B16-OVA TCL, poly I:C, Hiltonol and/or BO112 for 1 h, re-purified and plated with CellTrace Violet (CV)-labelled OT-I cells. Quantification (left) and representative histograms (right) of OT-I cell proliferation 3 days after. Combined data of 3 independent experiments. **d** cDC1s were cultured for 1 h with poly I:C, soluble OVA protein and/or B16-OVA TCL and 2 × 10^5^ cDC1s were injected intravenously (IV) into CD45.2+ recipient mice that had received 1-2 × 10^5^ CV-labelled CD45.1+ OT-I CD8+ T cells one-day prior. Equally treated “leftover” B16-OVA TCL served as negative control (Additional file 1: Figure S2d). Total OT-I cell number and number of IFNγ-producing OT-I cells after re-stimulation with OVA_257–264_ peptide was determined by flow cytometric quantification in spleen 5 days later. One representative of 2 independent experiments with n = 3 mice/group/experiment. **e** cDC1s were cultured for 1 h with poly I:C and B16-OVA TCL and 2 × 10^5^ cDC1s were IV or intradermally (ID) injected into CD45.2+ recipient mice that had received 1-2 × 10^5^ CD45.1+ OT-I CD8+ T cells one-day prior. Equally treated “leftover” B16-OVA TCL served as negative control (Additional file 1: Figure S2d). OT-I number and frequency was determined by flow cytometric quantification in spleen and draining lymph node (iLN) 5 days later. One representative of 2 independent experiments with *n* = 4 mice/group/experiment. *P < 0.05, **P < 0.01, ***P < 0.001 by **a** & **c**-**e** one-way ANOVA and Tukey post hoc test or **b** paired Student’s *t* test

Most Ag preparations and delivery regimes for induction of anti-tumor immunity have been optimized for in vitro-generated moDCs and may be not equally suitable for natural DC subsets with specific intrinsic functional properties. Utilization of a variety of different tumor-associated Ags is likely to enhance the efficacy of DCs to mount a general anti-cancer immune response and thereby limits the escape of individual tumor-Ag variant loss. Enticingly, ICD of tumor cells induced by methods such as UV irradiation induces strong DC-mediated immunity to dead-cell associated Ags [37, 38]. Therefore, we prepared syngeneic TCL of total mouse cancer cells by UV irradiation, followed by culture o/n to allow secondary necrosis and 3 freeze/thaw cycles to ensure cell death (Additional file 1: Figure S2a). UV irradiation induced release of high-mobility group box protein 1 (HMGB1), a hallmark of ICD, by OVA-expressing B16 melanoma cells (B16-OVA) [28], even higher than the strong ICD-inducer doxorubicin, whereas the non-ICD inducers mitomycin C and brefeldin A [38, 39] had no effect (Fig. 2a). ICD-mediated HMGB1 release results in DC activation and cross presentation [40, 41]. In line, natural spleen cDC1s treated ex vivo with B16-OVA TCL upregulated CD86 and MHC-II expression, which was dependent on soluble factors as washed TCL failed to do so (Fig. 2b and Additional file 1: Figure S2b). Addition of the potent stimulant poly I:C during TCL treatment further amplified their maturation, while CD86 levels remained slightly higher in TCL-treated cDC1s compared to controls (Fig. 2b and Additional file 1: Figure S2b). To determine optimal cDC1 activation, cDC1s were exposed to B16-OVA TCL in the presence of poly I:C (from InvivoGen), Hiltonol® (poly ICLC from Oncovir Inc., in clinical use) or BO-112 (a synthetic dsRNA complex targeting cytosolic helicases MDA5 and RIG-I as well as TLR3, in clinical trials: ClinicalTrials.gov identifier: NCT02828098). B16-OVA TCL-loaded and poly I:C-treated cDC1s showed the strongest potential to induce naive OT-I CD8+ T cell proliferation ex vivo, while the clinical-grade poly I:C analogues BO-112 and Hiltonol® were less effective (Fig. 2c). Hence, we focused on poly I:C as optimized adjuvant for primary mouse spleen cDC1s.

Next, we tested the efficiency of in vivo administration of cDC1s treated with B16-OVA TCL and poly I:C in mice adoptively transferred with OT-I CD8+ T cells. Notably, cDC1s treated with B16-OVA TCL and poly I:C induced increased total OT-I number and frequency as well as augmented IFNγ production upon OVA_257–264_ re-stimulation of splenocytes compared with poly I:C + soluble OVA-treated cDC1s and additional control treatments without cDC1s or without Ag (Fig. 2d and Additional file 1: Figure S2c & d). This observation pointed towards the efficacy of UV irradiation-induced TCL as an optimal Ag source for natural cDC1s fostering their activation.

Once established an optimal adjuvant-Ag combination, we tested administration of TCL-loaded cDC1s via intradermal (ID) or IV injection in OT-I transferred mice. IV injection of B16-OVA TCL-loaded cDC1s induced strong OT-I T cell responses in the spleen but minor effects in the iLN, while ID administration induced strong OT-I expansion both in the spleen and the draining iLN (Fig. 2e). This result suggested ID administration as the optimal injection route for TCL-loaded cDC1s for treatment of tumors growing in the skin such as melanoma.

### Tumor Ag-loaded cDC1s cross-prime endogenous CD8+ T cells

**Fig. 3:**
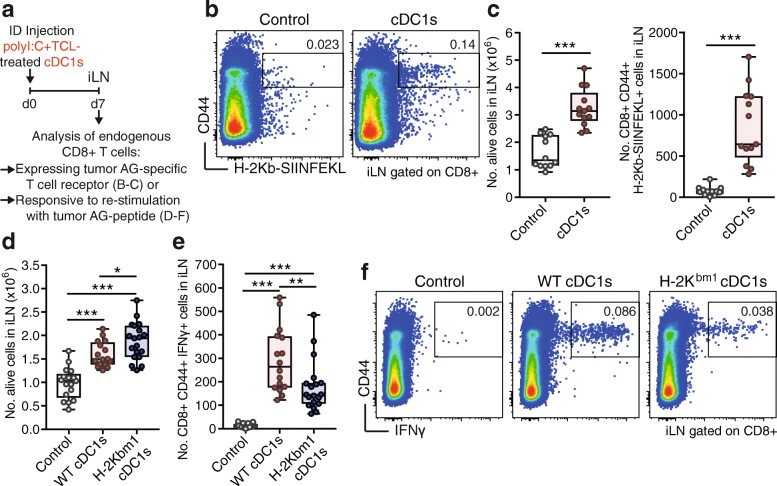
cDC1s cross-present tumor Ag to activate endogenous CD8+ T cells. **a** Schematic representation of experimental setup for data shown in (**b**-**f**). PBS control or 5 × 10^5^ poly I:C and B16-OVA TCL-loaded cDC1s were intradermally (ID) injected into mice and the draining inguinal lymph node (iLN) analyzed 7 days thereafter. **b** Representative plots and **c** (left panel) quantification by flow cytometry of total cell number and **c** (right panel) CD8+ CD44+ H-2Kb-SIINFEKL+ T cell number in iLN of mice ID injected with control PBS or 5 × 10^5^ poly I:C and B16-OVA TCL-loaded cDC1s. Combined data of 3 independent experiments with total *n* = 12 (Control) and *n* = 13 (cDC1s) mice are shown. ***P < 0.001 by Student’s *t* test. **d**-**f** Flow cytometric quantification of **d** total cell number and **e** CD8+ CD44+ IFNγ-producing cell number after re-stimulation with OVA_257–264_ peptide in iLN as well as **f** representative plots of mice ID injected with control PBS, 5 × 10^5^ poly I:C and B16-OVA TCL-loaded wildtype (WT) or H-2K^bm1^-harboring (H-2Kbm1) cDC1s. Combined data of 3 independent experiments with total *n* = 17 (Control), *n* = 16 (WT cDC1s) and *n* = 19 (H-2Kbm1 cDC1s) mice are shown. *P < 0.05, **P < 0.01, ***P < 0.001 by one-way ANOVA and Tukey post hoc test

We aimed to determine the potency of TCL-loaded cDC1s to stimulate effector responses from the endogenous repertoire of CD8+ T cells (Fig. 3a). Following ID administration of B16-OVA TCL-loaded cDC1s to naive mice, we found increased cellularity in the iLN, including augmented numbers of CD8+ T cells and higher frequency and numbers of activated OVA-specific (H-2Kb-OVA_257–264_+) CD8+ T cells (Fig. 3b & c and Additional file 1: Figure S3a). These data show that transferred TCL-loaded cDC1s prime endogenous CD8+ T cells. Of note, the whole TCL contains other Ags in addition to OVA, which would increase the total number of cDC1 vaccine-induced endogenous anti-tumor CD8+ T cells.

Next, we evaluated if the observed induction of endogenous CD8+ T cell responses in vivo was dependent on cross-presentation on MHC-I of the ex vivo acquired tumor-Ag by the transferred cDC1s. cDC1s were obtained from B16-FLT3L tumor-grafted WT and C57BL/6^H2Kbm1^ (H-2K^bm1^) mice, which harbor a mutation in the H-2K MHC-I molecule preventing the presentation of OVA_257–264_ peptide [42] (Fig. 3a). Adoptive transfer (ID) of TCL-loaded poly I:C-treated cDC1s from H-2K^bm1^ mice led to similar induction of total cellularity and amount of CD8+ T cells in the iLN compared with WT cDC1s (Fig. 3d and Additional file 1: Figure S3b). In contrast, transfer of cDC1s from H-2K^bm1^ mice resulted in reduced numbers and frequencies of activated CD44+ IFNγ-producing CD8+ T cells upon re-stimulation compared with WT cDC1s (Fig. 3e & f and Additional file 1: Figure S3b). These results indicate that TCL-loaded cDC1s are cross-presenting the ex vivo acquired tumor Ag to induce CD8+ T cell responses from the endogenous repertoire in vivo.

### Vaccination with TCL-loaded cDC1s protects against melanoma engraftment

**Fig. 4:**
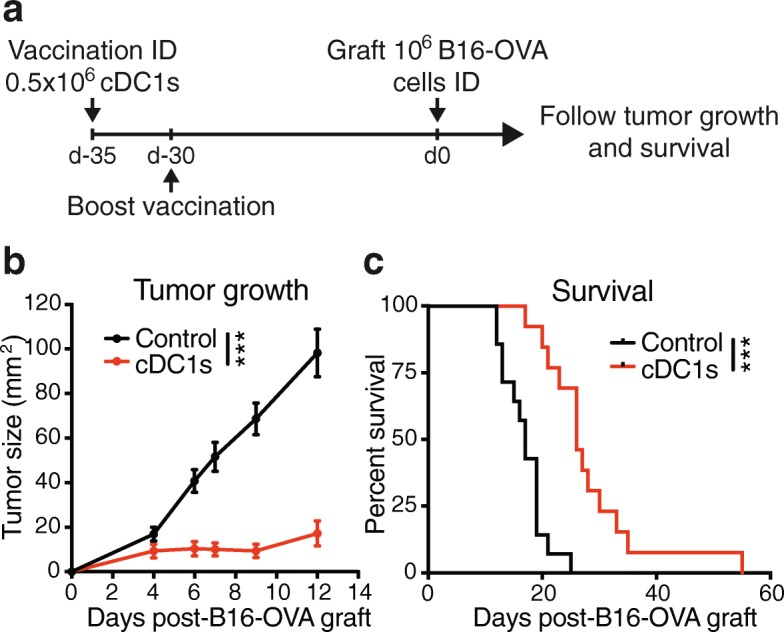
Vaccination with TCL-loaded cDC1s protects against melanoma engraftment. **a** Schematic representation of treatment and analysis. PBS control or 0.5 × 10^6^ poly I:C and autologous B16-OVA TCL-loaded cDC1s were intradermally (ID) injected 35 and 30 days prior to ID injection of 10^6^ B16-OVA cancer cells (day 0) and tumor growth and survival monitored. **b** Tumor growth and **c** survival curve of mice treated as described in (**a**). Combined data of 2 independent experiments with total *n* = 14 (Control) and *n* = 13 (cDC1s) mice are shown. ***P < 0.001 by **b** Two-way ANOVA or **c** Mantel-Cox test

Next, we investigated whether the TCL-loaded cDC1-induced tumor Ag-specific CD8+ T cell response would prevent a subsequent tumor challenge. To this end, we ID injected naive mice with B16-OVA TCL-loaded cDC1s, followed by a boost vaccination 5 days later and ID grafting of B16-OVA melanoma cells 30 days after vaccination (Fig. 4a). cDC1-vaccinated mice showed a marked reduction of tumor growth and improved survival compared with control mice (Fig. 4b & c). These data show that ID administration of TCL-loaded cDC1s induces long-lasting anti-tumor effects.

### Transfer of TCL-loaded cDC1s enhances tumor-Ag-specific T-cell presence in tumors and controls established cancer progression

**Fig. 5:**
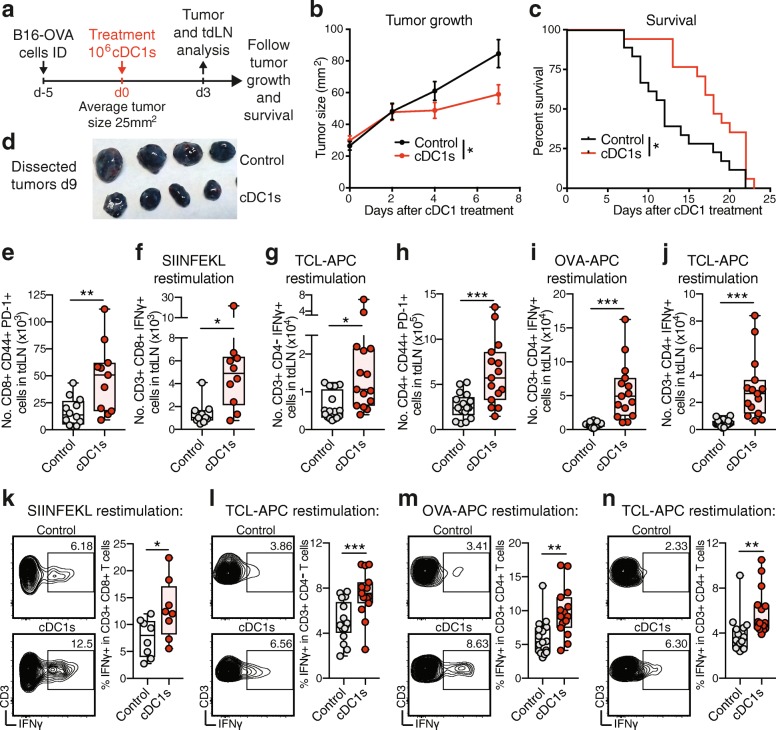
Transfer of TCL-loaded cDC1s enhances tumor-Ag-reactive T-cell presence in tumors and control established cancer progression. **a** Schematic representation of treatment and analysis for data shown in (**b**-**n**). Mice were intradermally (ID) injected with 10^6^ B16-OVA cancer cells followed by ID injection of PBS control or 10^6^ poly I:C and B16-OVA TCL-loaded cDC1s 5 days after, when tumors reached a size >10mm^2^ and < 55 mm^2^ (day 0). Either **b**-**d** tumor growth and survival were monitored or **e**-**n** animals sacrificed 3 days after cDC1 treatment for analysis of tumor and tumor-draining lymph node (tdLN). **b** Tumor growth and **c** survival curve. Combined data of 2 independent experiments with total *n* = 18 (Control) and *n* = 17 (cDC1s) mice are shown. *P < 0.05 by **b** Student’s *t* test on day 7 or **c** Mantel-Cox test. In a separate experiment, **d** tumors (*n* = 4) were dissected 9 days after cDC1 treatment and photographed. **e**-**j** Flow cytometric quantification of tdLN CD8+ T cells: **e** CD8+ CD44+ PD-1+, CD3+ CD8+ IFNγ+ or CD3+ CD4- IFNγ+ T cell number after re-stimulation with **f** OVA_257–264_ peptide or **g** B16-OVA TCL-loaded antigen-presenting cells (APCs) and tdLN CD4+ T cells: **h** CD4+ CD44+ PD-1+, CD3+ CD4+ IFNγ+ T cell number after re-stimulation with **i** OVA_323–339_ peptide-loaded APCs or **j** B16-OVA TCL-loaded APCs. **k**-**n** Flow cytometric quantification and representative histogram (gated on CD3+ and CD8+, CD4- or CD4+ cells) of tumor CD8+ T cells: CD3+ CD8+ IFNγ+ or CD3+ CD4- IFNγ+ T cell number after re-stimulation with **k** OVA_257–264_ peptide or **l** B16-OVA TCL-loaded APCs and tumor CD4+ T cells: CD3+ CD4+ IFNγ+ T cell number after re-stimulation with **m** OVA_323–339_ peptide-loaded APCs or **n** B16-OVA TCL-loaded APCs. Combined data of 2 independent experiments with total *n* = 12–17 (Control) and *n* = 11–15 (cDC1s) mice are shown. *P < 0.05, **P < 0.01, ***P < 0.001 by Student’s *t* test

In a subsequent approach, we aimed to analyze the efficacy of a cDC1-induced anti-cancer effector T cell response in a therapeutic cancer setting. To this end, we determined the potential of TCL-loaded cDC1s to limit progression of established orthotopic (ID) B16-OVA melanomas with an average size of 25mm^2^ (Fig. 5a). ID injection of B16-OVA TCL-loaded cDC1s halted tumor progression (Fig. 5b) and extended survival of tumor-bearing mice to > 30% enhanced median survival compared with control mice (Fig. 5c & d). The timing of the melanoma growth control suggested the generation of an anti-cancer effector T cell response, therefore we analyzed CD8+ and CD4+ T cells present in the tumor-draining iLN (tdLN) (Fig. 5e-j and Additional file 1: Figure S4) and the grafted B16-OVA tumor (Fig. 5k-n and Additional file 1: Figure S5) 3 days after cDC1 administration (Fig. 5a). Indeed, tumor-bearing mice treated with cDC1s exhibited larger tdLNs, and the number and frequency of PD-1-expressing CD44+ activated CD8+ and CD4+ T cells and H-2Kb- OVA_257–264_+ CD44+ CD8+ OVA-specific T cells was significantly enhanced in the tdLNs, while CD44+ OVA-specific CD4+ T cells remained unaltered (Fig. 5e & h and Additional file 1: Figure S4a-c, f-h, k & l). Notably, re-stimulation with OVA_257–264_ peptide, MHC class II OVA_323–339_ peptide-loaded APCs and/or B16-OVA TCL-loaded APCs of CD8+ and CD4+ T cells in tdLNs 3 days after TCL-loaded cDC1 injection resulted in strongly augmented IFNγ production (Fig. 5f, g, i & j and Additional file 1: Figure S4d, e, i & j).

We next determined T cell presence in the tumor 3 days after cDC1 treatment. At this time point, tumor size and frequency of total or PD-1-expressing CD8+ and CD4+ T cells, as well as OVA-specific CD4+ T cells in the tumor was equal (Additional file 1: Figure S5a-c & e-g). In contrast, the frequency of tumor Ag-specific H-2Kb- OVA_257–264_+ CD8+ T cells was strongly increased after TCL-loaded cDC1 treatment (Additional file 1: Figure S5d). Moreover, a significantly enhanced frequency of CD8+ and CD4+ T cells producing IFNγ upon re-stimulation with OVA_257–264_ peptide, OVA_323–339_ peptide-loaded APCs and/or B16-OVA TCL-loaded APCs were detected in tumors of cDC1 vaccinated mice (Fig. 5k-n). Our results establish the efficacy of ID-administered natural cDC1s loaded with syngeneic cell death-induced TCL for therapeutic treatment of established orthotopic melanoma. Mechanistically, administration of cDC1s increases tumor Ag-specific effector CD8+ T cells and recall responses of CD8+ and CD4+ T cells in the tdLN and the tumor short before the decrease in tumor growth becomes significant.

### Treatment with TCL-loaded cDC1 strongly improves anti-PD-1 therapy

**Fig. 6:**
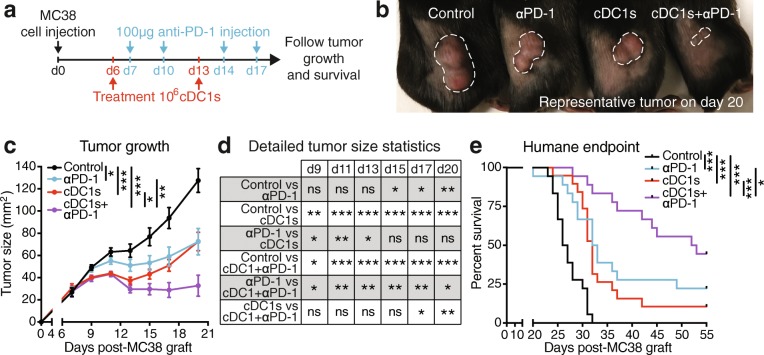
Whole tumor Ag-loaded natural cDC1s reduce progression of anti-PD-1 susceptible MC38 tumors, potentiating anti-PD-1 therapy. **a** Schematic representation of treatment and analysis. Mice grafted subcutaneously with 10^6^ MC38 cancer cells (day 0) were intradermally injected with PBS (Control and αPD-1-treated groups) or 10^6^ poly I:C and autologous MC38 TCL-loaded cDC1s (cDC1s- and cDC1s + αPD-1-treated groups) at day 6 & 13 days as well as intraperitoneally injected with PBS (Control and cDC1s-treated groups) or 100 μg anti-PD-1 antibody (αPD-1- and cDC1s + αPD-1-treated groups) on days 7, 10, 14 & 17 and tumor progression monitored. **b** Representative images of tumors on day 20, **c** tumor growth, **d** detailed statistics of tumor size at individual days and **e** survival curve before reaching the humane endpoint of mice treated as described in (**a**). Remaining, surviving mice in (**e**) completely rejected the tumor and were followed for at least 3 months. White dashed lines in (**b**) indicate tumor margin. Combined data of 2 independent experiments with total *n* = 18 (Control, αPD-1 and cDC1s + αPD-1-treated groups) and n = 19 (cDC1s-treated group) mice are shown. *P < 0.05, **P < 0.01, ***P < 0.001 by **c** Two-Way ANOVA, *P < 0.05, **P < 0.01, ***P < 0.001 by **d** Student’s *t* test, *P < 0.05, ***P < 0.001 by **e** Mantel-Cox test, ns: not significant

In order to put the treatment with TCL-loaded natural cDC1s into the context of current therapies, we compared and combined it with immune checkpoint inhibition. MC38 colon adenocarcinoma, a wildtype tumor model that does not express any exogenous or dominant Ags, was chosen as a tumor partially susceptible to anti-PD-1 therapy when grafted subcutaneously [28]. UV irradiation of MC38 cancer cells induced HMGB1 release (Additional file 1: Figure S6a). Moreover, ex vivo cDC1 treatment with UV irradiation-generated MC38 TCL resulted in upregulation of CD86 and MHC-II, however not further increasing poly I:C-mediated cDC1 activation (Additional file 1: Figure S6b & c). MC38 tumor-bearing mice were treated with cDC1s loaded with MC38 TCL when tumors were visible (day 6) and 1 week later (day 13). Further, mice received intraperitoneal (IP) injections of anti-PD-1 antibody on days 7, 10, 14 and 17 (Fig. 6a). MC38 TCL-loaded cDC1 administration was overall equally efficient as anti-PD-1 therapy in decreasing MC38 tumor progression and extending survival of mice before reaching the humane endpoint compared with control PBS treatment (Fig. 6b-e and Additional file 1: Figure S6d). Interestingly, cDC1-treated mice showed a faster therapeutic effect (Fig. 6c & d), despite almost equal timing of treatment start and more frequent administration of anti-PD-1 antibody (Fig. 6a). Notably, combined treatment of MC38 tumor-bearing mice with TCL-loaded cDC1s and anti-PD-1 was more effective than single treatments, with complete tumor rejection in 8/18 mice (Fig. 6b-e and Additional file 1: Figure S6d). These results support that adoptive immunotherapy with TCL-loaded natural cDC1s improves anti-PD-1 treatment, significantly extending survival and doubling the number of mice cured from grafted tumors.

### Immunotherapy with TCL-loaded cDC1s extends survival of anti-PD-1-refractory B16/F10 melanoma-bearing mice

**Fig. 7:**
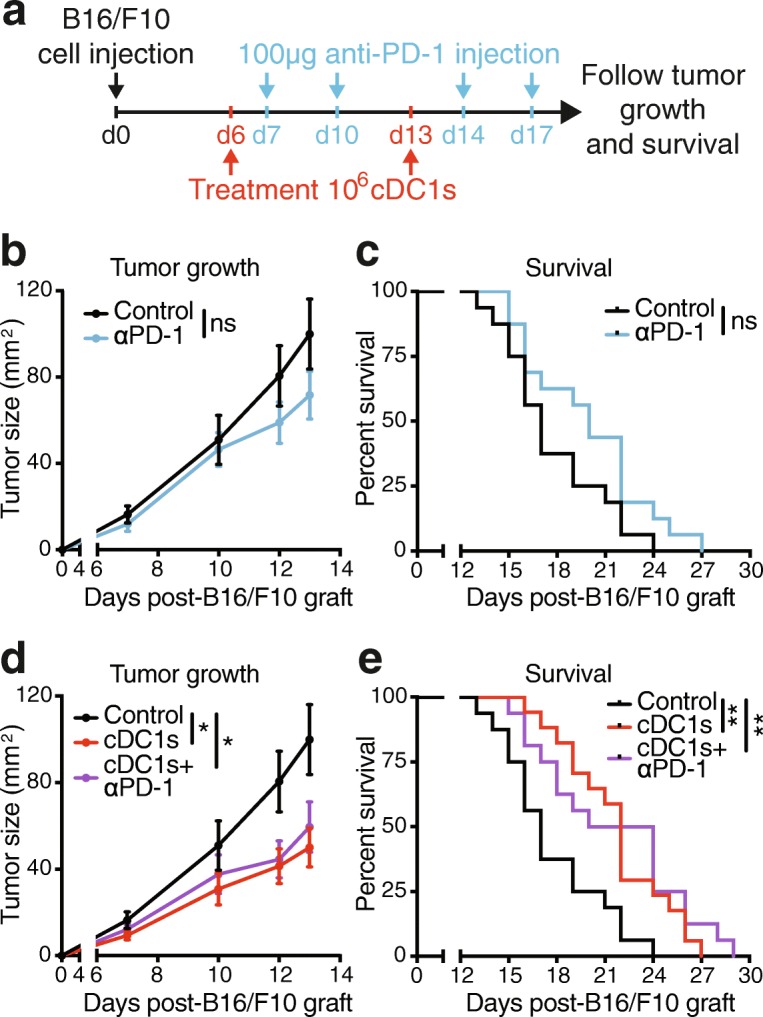
Cell death-induced tumor Ag-loaded cDC1s are effective for treatment of largely anti-PD-1 refractory B16/F10 melanoma. **a** Schematic representation of treatment and analysis. Mice grafted intradermally (ID) with 5 × 10^5^ B16/F10 cancer cells (day 0) were ID injected with PBS control (Control and αPD-1-treated groups) or 10^6^ poly I:C and autologous B16/F10 TCL-loaded cDC1s (cDC1s- and cDC1s + αPD-1-treated groups) at day 6 & 13 as well as intraperitoneally injected with PBS control (Control and cDC1s-treated groups) or 100 μg anti-PD-1 antibody (αPD-1- and cDC1s + αPD-1-treated groups) at day 7, 10, 14 & 17 and tumor growth and survival monitored. **b** & **d** Tumor growth and **c** & **e** survival curve of mice treated as described in (**a**). The same control group (black) is presented in (**b** & **d**) as well as in (**c** & **e**), because data belong to the same experiment and are split for clarity. The graphs are displayed separately for better visibility. Combined data of 2 independent experiments with total *n* = 16 (Control, αPD-1 and cDC1s + αPD-1-treated groups) and n = 17 (cDC1s-treated group) mice are shown. *P < 0.05 by **b** & **d** Student’s *t* test at day 12 and 13 and **P < 0.01 by **c** & **e** Mantel-Cox test, ns: not significant

We next tested therapeutic efficacy of cDC1 administration in established ID engrafted B16/F10 melanoma, a very aggressive tumor that does not express exogenous or dominant Ags and is largely refractory to anti-PD-1 therapy [28, 43]. UV irradiation also induced ICD of B16/F10 melanoma cells, as indicated by HMGB1 release and upregulation of MHC-II and CD86, which remained higher even upon poly I:C addition, on cDC1s exposed to B16/F10 TCL (Additional file 1: Figure S6e-g). Natural cDC1s loaded with syngeneic B16/F10 TCL were ID injected 6 and 13 days after grafting of B16/F10-melanomas onto recipient mice, when tumors were clearly visible. Anti-PD-1 antibody was IP injected on day 7, 10, 14 and 17 (Fig. 7a) and cancer progression monitored. As expected, mono-therapy with anti-PD-1 antibody did not significantly alter B16/F10 tumor growth or survival as compared with control PBS administration (Fig. 7b & c). Notably, treatment with B16/F10 TCL-loaded cDC1s reduced progression of established B16/F10 melanomas as compared with control mice, while combined administration of anti-PD-1 and TCL-loaded cDC1s mirrored the efficacy of cDC1 mono-treatment (Fig. 7d & e). These results suggest that anti-PD-1-refractory cancers could benefit from immunotherapy with ICD-induced TCL-loaded natural cDC1s.

## Discussion

The superior ability of mouse cDC1s to transport tumor-Ag to LNs, cross-present cancer cell-associated Ags and mediate infiltration of T cells in the tumor is established [14, 22, 29–31]. However, the efficiency of adoptive transfer of syngeneic natural circulating cDC1s for immunotherapy of cancer has not been previously tested. This study reveals the efficacy and feasibility of cancer-Ag loaded naturally occurring mouse cDC1s for treatment of cancer. This next-generation DC vaccine employs: (A) the conventional cDC1 subset purified from tumor-bearing mice that excels in cross priming and elicits the strongest anti-cancer CD8+ T cell responses [22, 44]; (B) the use of poly I:C as an adjuvant that targets cDC1s via TLR3 and induces immunogenic activation, equivalent to adjuvants currently tested for clinical use [27] (ClinicalTrials.gov identifier: NCT02828098); (C) cDC1-loading with a total tumor Ag preparation based on UV irradiation-induced ICD of cancer cells, a method reported to exhibit superior immunogenic potential [35, 37, 45, 46], releasing HMGB1 and activating cDC1s upon exposure; (D) a convenient route of administration; and (E) a time-efficient, economic and easy-to-use preparation protocol with translational potential.

FLT3L-mediated expansion of DCs facilitated isolation of mouse CD8+ cDC1s from the spleen of tumor-bearing mice. Amplification of DCs by administration of FLT3L in cancer patients is well-tolerated and might even have anti-cancer immunotherapeutic effects by itself in humans and mice [9, 29, 47]. Separation of natural CD1c + cDCs (cDC2s) and BDCA4+ pDCs from human leukapheresis products without the need for fluorescence-activated cell sorting and in good manufacturing practice (GMP) conditions was already achieved [16, 17] and technological advances will hopefully soon make GMP isolation of human BDCA3+ cDC1s possible (PROCROP Cancer Immunotherapy European Initiative Web Site. http://www.procrop.eu (2015); accessed 25 Feb 2019).

Herein, we establish the proof of principle that mouse natural cDC1s treated ex vivo with adjuvant and tumor-Ag for a very short time are efficient to induce Ag-specific anti-cancer CD8+ and CD4+ T cell responses in vivo. UV irradiation followed by secondary necrosis and generation of TCL from three different cancer cell lines served as source of immunogenic tumor cell Ag for cDC1s. Loading of cDC1s with cell death-induced TCL caused much more effective induction of CD8+ T cell activation than loading cDC1s with soluble protein. This result highlights that UV irradiation-induced TCL not only contains a plethora of tumor cell-associated Ags, but also danger signals like HMGB1 that activate cDC1s and their capacity for tumor Ag processing [35, 37, 40, 41, 45, 46]. Indeed, some cytostatic agents, such as anthracyclines and UV irradiation, induce ICD associated with a spatiotemporal release of danger-associated molecular patterns (such as calreticulin, ATP, HMGB1 and F-actin). Thereby, in contrast to apoptosis that has rather tolerogenic effects, ICD acts as potent stimulator of adaptive immunity, which depends on the presence of DCs [37–41, 45]. RIPK1 signaling and NF-κB-induced transcription in dying tumor cells also increase cross-priming efficiency and anti-tumor immunity [48]. Cell-associated danger signals associated with ICD like calreticulin promote phagocytosis, while adjuvants such as HMGB1 or F-actin induce DC activation, tumor Ag processing and cross-presentation [40, 41, 45, 49]. This is consistent with our results showing that UV irradiation causes HMGB1 release from three different mouse cancer cells lines and soluble factors in the resulting TCL induce DC activation. Moreover, treatment of natural cDC1s with both poly I:C adjuvant and TCL derived from UV-dead cells is needed for optimal CD8+ T cell cross-priming. Accordingly, a recent study showed that the combination of poly I:C activation and provision of necrotic cell-associated material caused human BDCA3+ cDC1s to outperform CD1c+ cDC2 and moDC subsets in Ag uptake, internalization and cross-presentation [50].

The here reported next-generation cDC1-based anti-cancer vaccine strongly induced anti-cancer effector CD8+ T cell responses from the endogenous repertoire in the skin-draining LN. Those responses appear to largely depend on the MHC class I-mediated presentation of the Ag loaded ex vivo onto cDC1s, because H-2K^bm1^ cDC1s, which fail to efficiently present OVA_257–264_ peptide on MHC-I [42], were significantly less potent than WT cDC1s. Notwithstanding, vaccination with H-2K^bm1^-harboring cDC1s induced some degree of specific CD8+ T cell response, suggesting that cDC1s may also transfer ex vivo obtained Ag to endogenous Ag presenting cells upon injection in vivo, consistent with a role of cDC1s in tumor Ag transport to tdLNs [29, 30].

CD8+ and CD4+ T cell activation induced by administration of TCL-loaded cDC1s contributes to reduced tumor progression and even remission of established tumors derived from the three cancer cell lines B16-OVA, B16/F10 and MC38, the latter two not expressing exogenous Ags. The effector tumor Ag-specific and cancer-reactive CD8+ and CD4+ T cell response from the endogenous repertoire is enhanced both in the tumor and tdLN and precedes delays in tumor growth.

Moreover, our next-generation cDC1 anti-cancer vaccine was also effective in a preventive cancer setting, suggesting induction of enduring immune responses and probably the formation of immune memory, which would indicate a potential efficacy preventing metastasis. Moreover, cDC1s were shown to contribute to optimal generation of tissue-resident memory CD8+ T cells [51], a memory subset that promotes anti-tumor immunity in concert with circulating memory CD8+ T cells [52]. This observation may have implications for adjuvant vaccination in postsurgical minimal residual disease settings.

Anti-PD-1 therapy is the current standard treatment for many cancer types [3, 4]. Efficacy of therapy with PD-1 blockade in mouse cancer models is dependent on cDC1s [28, 29]. In addition, we observe that administration of TCL-loaded cDC1s increased PD-1+ CD8+ and CD4+ T cell numbers in tdLNs. Therefore, it was important to compare and combine our next-generation cDC1 anti-cancer vaccine with anti-PD-1 blockade. Tumor Ag-loaded cDC1 mono-therapy was equally successful as anti-PD-1 treatment in grafted MC38 tumors. Notably, cDC1 administration was immediately effective, suggesting that it enlarges the pool of anti-cancer T cells which could explain the synergism of combination therapy with anti-PD-1 blockade in this model. Administration of TCL-loaded cDC1 is also effective in B16/F10 tumors, which are anti-PD-1-refractory [28]. This result supports the notion that DC-based cancer immunotherapy can act in synergy and go beyond checkpoint antibody therapy, which is crucial to improve treatment for tumors resistant to current strategies [4, 8, 22].

We have established the proof of principle showing that adoptive transfer of TCL-loaded and adjuvant-activated cDC1s is effective in cancer immunotherapy. However, we intentionally did not compare this therapy with administration of other DC subsets in the mouse system. Apart from the only clinically approved Sipuleucel-T treatment [7], there is currently no gold standard for DC therapy regarding DC subset, incubation time, Ag and/or adjuvant that would serve for comparison in humans [10], and less so in mice. Moreover, while cDC1s specialize on cross-presentation of cell-associated Ags in the mouse, the unique functions of the human cDC1 equivalents are also debated [15, 18–22]. Notably, analysis of several datasets including The Cancer Genome Atlas (TCGA) suggest that a cDC1 signature within many human tumors is associated with improved survival [26, 30, 32, 33]. Nonetheless, anti-cancer effects of DC-based vaccines will likely depend on numerous factors, including the nature of potential Ags available for a certain tumor type (identified neoantigens or tumor peptides, etc.) and, hence, the Ag and adjuvant cocktail used to load DCs. In addition, different tumors may benefit from generation of distinct types of immunity: for instance, a cDC2-dependent Th17 response may be beneficial in certain cancer models [53].

## Conclusions

Our proof-of-principle study provides, for the first time, pre-clinical data showing suitability and efficacy of therapeutic cross-presenting cDC1-based vaccination. Overall, our results suggest a feasible scenario for patient treatment based on administration of tumor-Ag-loaded cDC1s. We show efficacy of a cDC1-based therapy that does not require identification of tumor neoantigens, representing an easily achievable personalized Ag source for virtually any resected tumor. Circulating cDC1s isolated from leukapheresis products of patients, eventually undergoing additional treatments with FLT3L to enhance cDC1 numbers, can then be shortly exposed to autologous tumor-Ag and adjuvant before reinfusion. This strategy would reduce long-term cell cultures that make the process labor intensive and reduce reproducibility. Our results show the potential of this cDC1-based vaccine to be combined with standard anti-PD-1 therapy [54] or used in anti-PD-1 resistant tumors, inducing both effector and long-lasting anti-tumor CD8+ and CD4+ T cell responses. These prospects will be tested as soon as GMP isolation of human natural cDC1s is feasible.

## Additional file

Additional file 1: **Figure S1.** Isolation and phenotypic analysis of cDC1s from the spleen of FLT3L-expressing B16 tumor-bearing mice. **Figure S2.** Syngeneic TCL preparation for cDC1 maturation and cDC1-specific induction of T cell activation in vivo. **Figure S3.** Adoptive cDC1 transfer-mediated endogenous CD8+ T cell responses are dependent on presentation on MHC-I of tumor Ag loaded onto cDC1s. **Figure S4.** Analysis of T cells in tumor-draining lymph node after administration of tumor Ag-loaded cDC1s. **Figure S5.** Analysis of T cells in tumor after administration of tumor Ag-loaded cDC1s. **Figure S6.** UV irradiation-induced ICD of MC38 and B16/F10 cancer cells results in HMGB1 release and cDC1 activation. (PDF 3157 kb)

## Abbreviations

Ag: Antigen
B16-FLT3L: FLT3L-expressing B16 melanoma cells
B16-OVA: OVA-expressing B16 melanoma cells
CD8+ CTLs: Cytotoxic CD8+ T lymphocytes
cDC1: Conventional type 1 DC
cDC2: Conventional type 2 DC
DC: Dendritic cell
FLT3L: FMS-like tyrosine kinase 3 ligand
GMP: Good manufacturing practice
H-2K^bm1^ mice: C57BL/6^H2Kbm1^ mice
HMGB1: High-mobility group box protein 1
ICD: Immunogenic cell death
ID: Intradermal
iLN: Inguinal LN
IP: Intraperitoneal
ISQAVHAAHAEINEAGR: OVA_323–339_ peptide
IV: Intravenous
LN: Lymph node
moDCs: Monocyte-derived DCs
o/n: Overnight
OVA: Ovalbumin
pDC: Plasmacytoid DC
SIINFEKL: OVA_257–264_ peptide
TCL: Tumor cell lysate
tdLN: Tumor-draining iLN
TLR3: Toll-like receptor 3
UV: Ultraviolet
