# Supplementary material for: Effective cancer immunotherapy by natural mouse conventional type-1 dendritic cells bearing dead tumor antigen
Source: J Immunother Cancer. 2019 Apr 8;7:100. doi: 10.1186/s40425-019-0565-5 (PMC6454603; doi:10.1186/s40425-019-0565-5)
Supplement: Supplementary file 1 — Figure S1. Isolation and phenotypic analysis of cDC1s from the spleen of FLT3L-expressing B16 tumor-bearing mice. Figure S2. Syngeneic TCL preparation for cDC1 maturation and cDC1-specific induction of T cell activation in vivo. Figure S3. Adoptive cDC1 transfer-mediated endogenous CD8+ T cell responses are dependent on presentation on MHC-I of tumor Ag loaded onto cDC1s. Figure S4. Analysis of T cells in tumor-draining lymph node after administration of tumor Ag-loaded cDC1s. Figure S5. Analysis of T cells in tumor after administration of tumor Ag-loaded cDC1s. Figure S6. UV irradiation-induced ICD of MC38 and B16/F10 cancer cells results in HMGB1 release and cDC1 activation. (PDF 3157 kb) [file 40425_2019_565_MOESM1_ESM.pdf]

Effective cancer immunotherapy by natural mouse conventional type-1 dendritic cells bearing dead tumor antigen

Stefanie K. Wculek[1], Joaquín Amores-Iniesta[1], Ruth Conde-Garrosa[1], Sofía C. Khouili[1], Ignacio Melero[2,3,4] and David Sancho[1,*].

Additional file 1

- Supplemental Figures S1-S6
- Supplemental Figure legends

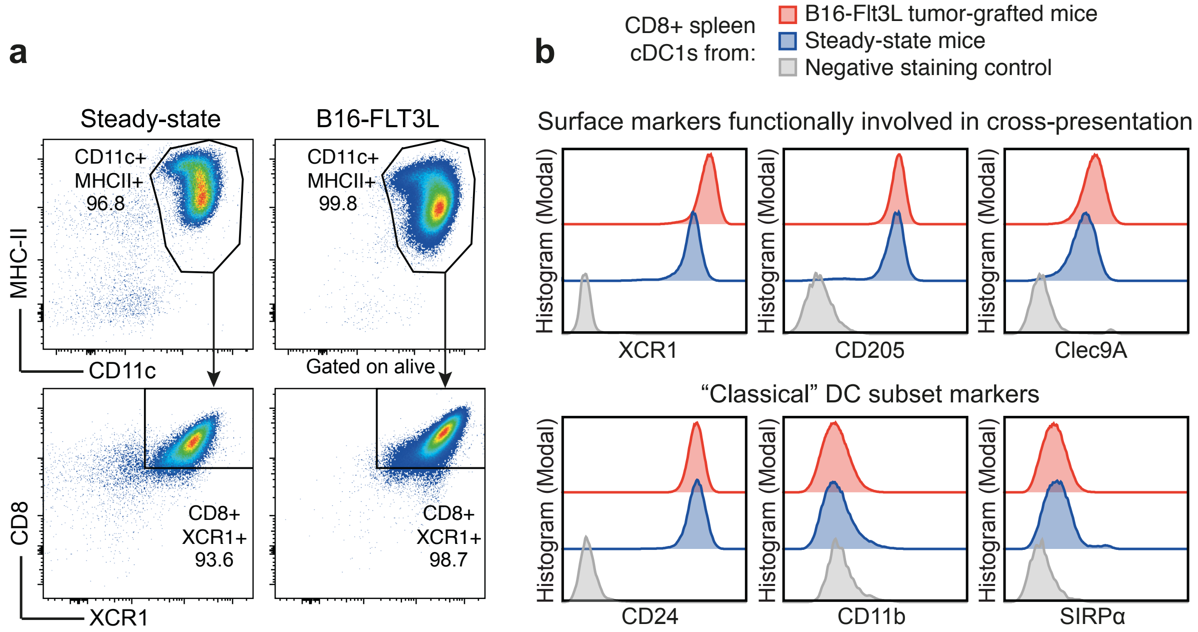

Figure S1. Isolation and phenotypic analysis of cDC1s from the spleen of FLT3L-expressing B16 tumor-bearing mice.

a & **b** CD8+ cDC1s were isolated from steady-state mice or mice harboring subcutaneous B16-FLT3L tumors as described in Methods. Yield was about 10^5 cells/steady-state spleen and 1-$5x10^6$ cells/B16-Flt3L-expanded spleen. **a** Representative flow cytometric analysis of cell purity of cDC1 preparation. Isolated cells were gated on alive (upper panel) and CD11c+ MHC-II+ cells (lower panel). **b** Representative flow cytometric analysis of cell surface marker expression on isolated CD11c+ MHC-II+ CD8+ cells. Antibody mix missing one antibody was used as negative staining control. One representative of ≥2 independent experiments is shown.

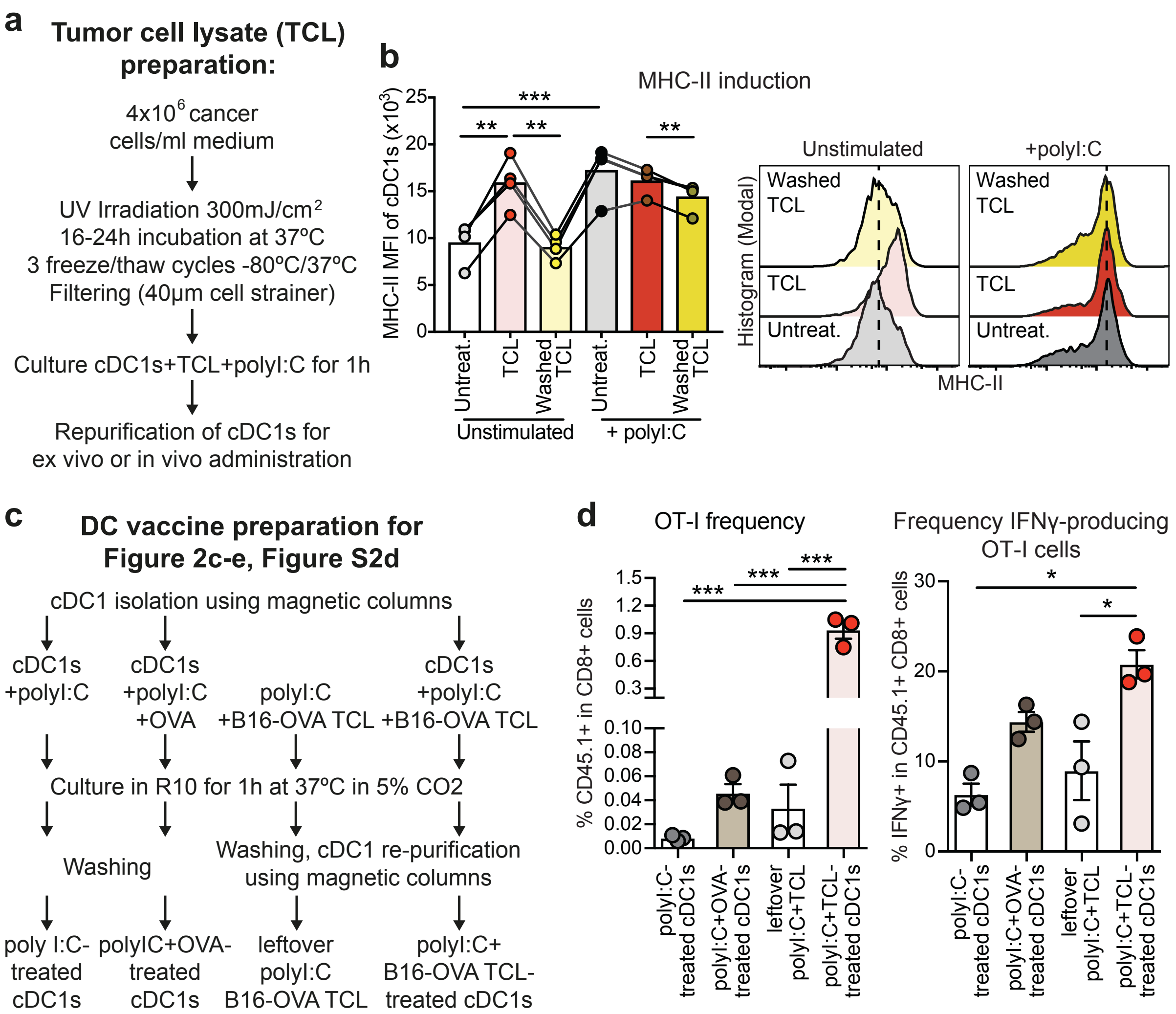

Figure S2. Syngeneic TCL preparation for cDC1 maturation and cDC1-specific induction of T cell activation in vivo.

a Overview of preparation of tumor cell lysate (TCL). **b** Quantification (left) and representative histograms (right) of MHC-II expression on untreated cDC1s, cDC1s treated with B16-OVA TCL or washed B16-OVA TCL containing only cellular components under absence or presence of 20µg/ml poly I:C. After 1 hour, cDC1s were washed and cultured for 4 hours followed by flow cytometric analysis. Combined data of 4 independent experiments are shown. **P<0.01, ***P<0.001 by paired Student's *t* test. MFI, mean fluorescence intensity. **c** Overview of treatments for preparation of cDC1 vaccines used in Figure 2c-e and Figure S2d. **d** Corresponding to Fig. 2d: cDC1s treated for 1h with 20µg/ml poly I:C, 20µg/ml soluble OVA protein and/or B16-OVA TCL at a ratio of 1:2 cDC1s versus tumor cells were washed and re-purified using magnetic separation (Miltenyi). 2x10^5 cDC1s were injected intravenously into CD45.2+ recipient mice that had been adoptively transferred with 1-2x10^5 CellTrace Violet (CV)-labelled CD45.1+ OT-I CD8+ T cells one-day prior and the spleen was harvested 5 days later. OT-I cell frequency and frequency of IFNγ-producing OT-I cells after re-stimulation with $OVA_{257-264}$ peptide in total CD8+ cells was determined by flow cytometric quantification. One representative of 2 independent experiments with n=3 mice/group/experiment is shown. *P<0.05, ***P<0.001 by one-way ANOVA and Tukey post hoc test.

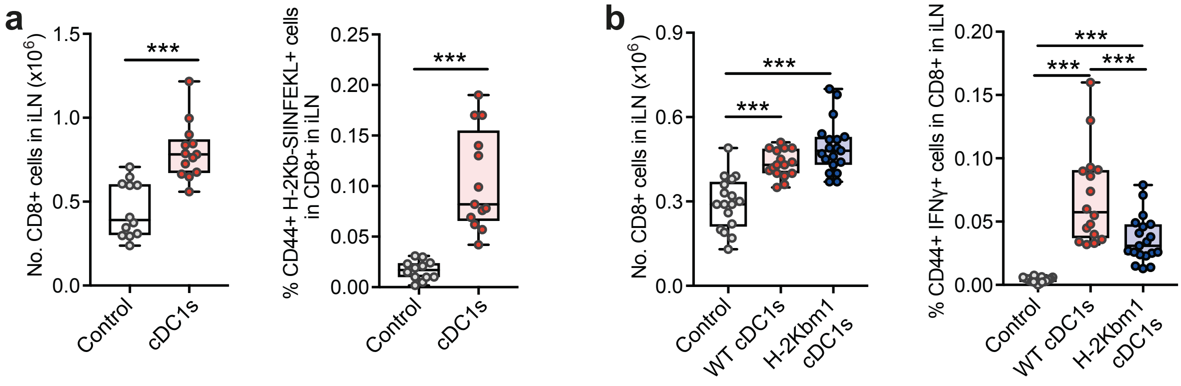

Figure S3. Adoptive cDC1 transfer-mediated endogenous CD8+ T cell responses are dependent on presentation on MHC-I of tumor Ag loaded onto cDC1s.

a Corresponding to Fig. 3b & c. Flow cytometric quantification of total CD8+ T cell number (left panel) and frequency of CD44+ H-2Kb-SIINFEKL+ within CD8+ T cells (right panel) in iLN 7 days after ID injection of control PBS or $5x10^5$ poly I:C and B16-OVA TCL-loaded cDC1s. Combined data of 3 independent experiments with total n=12 (Control) and n=13 (cDC1s) mice are shown. ***P<0.001 by Student's *t* test. **b** Corresponding to Figure 3d-f. Flow cytometric quantification of total CD8+ T cell number (left panel) and frequency of CD44+ IFNγ-producing cells after re-stimulation with $OVA_{257-264}$ peptide within CD8+ T cells (right panel) in iLN 7 days after ID injection of control PBS, $5x10^5$ poly I:C and B16-OVA TCL-loaded wildtype (WT) cDC1s or $H\text{-}2K^{bm1}$-harboring cDC1s. Combined data of 3 independent experiments with total n=17 (Control), n=16 (WT cDC1s) and n=19 (H-2Kbm1 cDC1s) mice are shown. ***P<0.001 by one-way ANOVA and Tukey post hoc test.

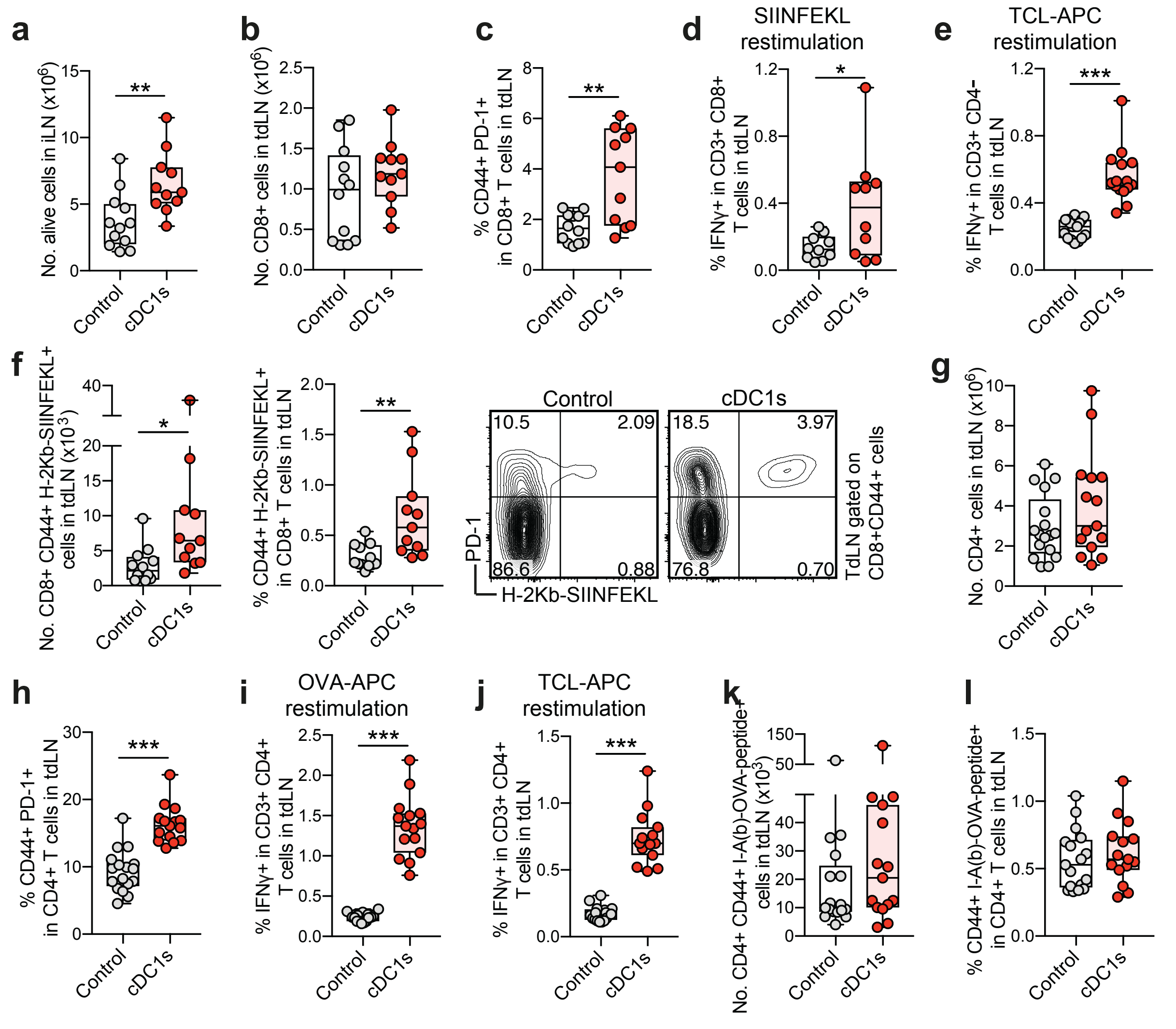

Figure S4. Analysis of T cells in tumor-draining lymph node after administration of tumor Ag-loaded cDC1s.

Corresponding to Fig. 5. B16-OVA tumor-bearing mice were intradermally injected with PBS control or 10^6 poly I:C and B16-OVA TCL-loaded cDC1s and T cell response in the tumor-draining lymph node (tdLN) analyzed 3 days thereafter. Flow cytometric quantification of **a** total tdLN cell number, **b** CD8+ T cell number, **c** frequency of CD44+ PD-1+ in CD8+ T cells, frequency of IFNγ+ in CD3+ CD8+ or CD3+ CD4- T cells after re-stimulation with **d** $OVA_{257-264}$ peptide or **e** B16-OVA TCL-loaded antigen-presenting cells (APCs), **f** CD8+ CD44+ H-2Kb-SIINFEKL+ T cell number and frequency with representative flow cytometric analysis (gated on CD8+ CD44+ cells), **g** CD4+ T cell number, **h** frequency of CD44+ PD-1+ in CD4+ T cells, frequency of IFNγ+ in CD3+ CD4+ T cells after re-stimulation with **i** $OVA_{323-339}$ peptide-loaded APCs or **j** B16-OVA TCL-loaded APCs, **k** number and **l** frequency of CD4+ CD44+ T cells positive for a OVA-specific MHC-II tetramer mix as indicated in Methods. Combined data of 2 independent experiments with total n=12-17 (Control) and n=11-15 (cDC1s) mice are shown. *P<0.05, **P<0.01, ***P<0.001 by Student's *t* test.

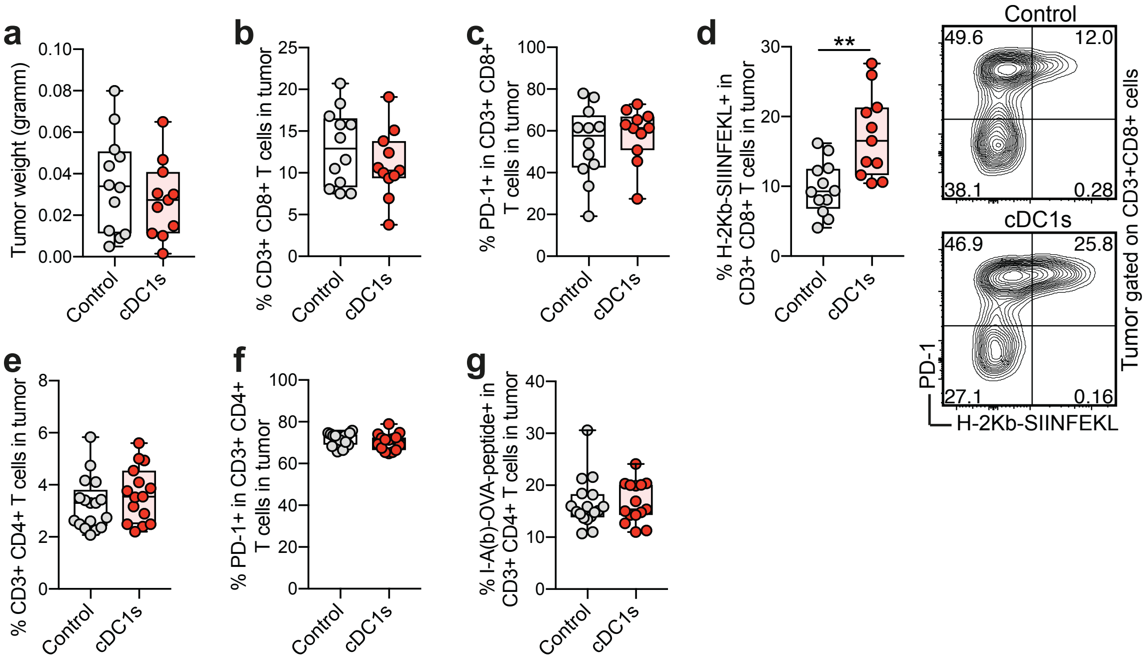

Figure S5. Analysis of T cells in tumor after administration of tumor Ag-loaded cDC1s. Corresponding to Fig. 5. B16-OVA tumor-bearing mice were intradermally injected with PBS control or 10^6 poly I:C and B16-OVA TCL-loaded cDC1s and T cell response in the tumor analyzed 3 days thereafter. **a** Quantification of tumor weight, flow cytometric quantification of **b** total CD3+ CD8+ T cell frequency, **c** frequency of PD-1+ in CD3+ CD8+ T cells, **d** frequency of H-2Kb- SIINFEKL+ in CD3+ CD8+ T cells with representative flow cytometric analysis (gated on CD3+ CD8+ cells), **e** total CD3+ CD4+ T cell frequency, **f** frequency of PD-1+ in CD3+ CD4+ T cells and **g** frequency of CD3+ CD4+ T cells positive for a OVA-specific MHC-II tetramer mix as indicated in Methods. Combined data of 2 independent experiments with total n=12-17 (Control) and n=11-15 (cDC1s) mice are shown. *P<0.05, **P<0.01, ***P<0.001 by Student's *t* test.

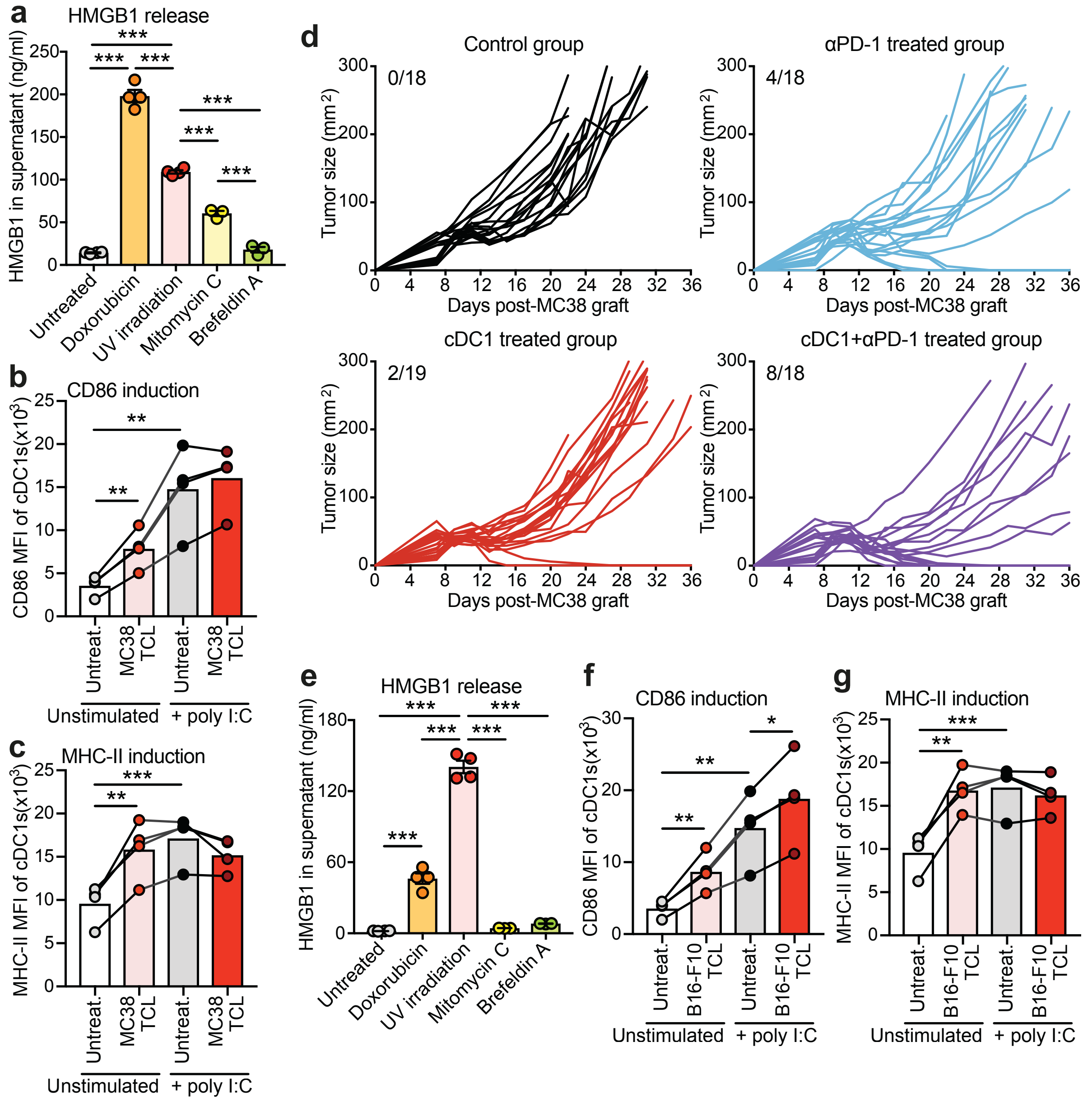

Figure S6. UV irradiation-induced ICD of MC38 and B16/F10 cancer cells results in HMGB1 release and cDC1 activation.

a-d Corresponding to Fig. 6 and **e-g** corresponding to Fig. 7. **a** & **e** HMGB1 content measured by ELISA in supernatants of **a** MC38 and **e** B16/F10 cells treated with 25µM doxorubicin, 300mJ/cm^2 UV irradiation, 30µM Mitomycin C or 50µM Brefeldin A and cultured for 18hrs (n=3-4). ***P<0.001 by one-way ANOVA and Tukey post hoc test. **b**, **c**, **f** & **g** Quantification of **b** & **f** CD86 and **c** & **g** MHC-II expression on untreated cDC1s (Untreat.), cDC1s treated for 1h with **b** & **c** MC38 tumor cell lysate (TCL) or **f** & **g** B16/F10 TCL in presence or absence of 20µg/ml poly I:C. cDC1s were washed and cultured for 4 hours followed by flow cytometric analysis. The same untreated control groups (white and gray) are presented in **b** & **f** as well as in **c** & **g**, because data belong to the same experiments and are split for clarity. Combined data of 4 independent experiments. *P<0.05, **P<0.01, ***P<0.001 by paired Student's *t* test. MFI, mean fluorescence intensity. **d** Individual tumor growth of mice subcutaneously grafted with 10^6 MC38 cancer cells followed by intradermal injection of PBS (Control and αPD-1-treated

groups) or 10^6 poly I:C and autologous MC38 TCL-loaded cDC1s (cDC1s- and cDC1s+αPD-1-treated groups) at day 6 & 13 days as well as intraperitoneal injection of PBS (Control and cDC1s-treated groups) or 100μg anti-PD-1 antibody (αPD-1- and cDC1s+αPD-1-treated groups) at day 7, 10, 14 & 17. Number of animals that rejected the tumor out of total mice/group are indicated in the upper left corner in every graph. Combined data of 2 independent experiments with total n=18 (Control, αPD-1 and cDC1s+αPD-1-treated groups) and n=19 (cDC1s-treated group) mice are shown.
